# NR4A2 Is Regulated by Gastrin and Influences Cellular Responses of Gastric Adenocarcinoma Cells

**DOI:** 10.1371/journal.pone.0076234

**Published:** 2013-09-27

**Authors:** Kristine Misund, Linn-Karina Myrland Selvik, Shalini Rao, Kristin Nørsett, Ingunn Bakke, Arne K. Sandvik, Astrid Lægreid, Torunn Bruland, Wenche S. Prestvik, Liv Thommesen

**Affiliations:** 1 Department of Cancer Research and Molecular Medicine, Norwegian University of Science and Technology (NTNU), Trondheim, Norway; 2 Faculty of Technology, Sør-Trøndelag University College, Trondheim, Norway; 3 Department of Gastroenterology and Hepatology, Medical Clinic, St. Olav’s University Hospital, Trondheim, Norway; Vanderbilt University Medical Center, United States of America

## Abstract

The peptide hormone gastrin is known to play a role in differentiation, growth and apoptosis of cells in the gastric mucosa. In this study we demonstrate that gastrin induces Nuclear Receptor 4A2 (NR4A2) expression in the adenocarcinoma cell lines AR42J and AGS-G_R_, which both possess the gastrin/CCK2 receptor. *In vivo*, NR4A2 is strongly expressed in the gastrin responsive neuroendocrine ECL cells in normal mucosa, whereas gastric adenocarcinoma tissue reveals a more diffuse and variable expression in tumor cells. We show that NR4A2 is a primary early transient gastrin induced gene in adenocarcinoma cell lines, and that NR4A2 expression is negatively regulated by inducible cAMP early repressor (ICER) and zinc finger protein 36, C3H1 type-like 1 (Zfp36l1), suggesting that these gastrin regulated proteins exert a negative feedback control of NR4A2 activated responses. FRAP analyses indicate that gastrin also modifies the nucleus-cytosol shuttling of NR4A2, with more NR4A2 localized to cytoplasm upon gastrin treatment. Knock-down experiments with siRNA targeting NR4A2 increase migration of gastrin treated adenocarcinoma AGS-G_R_ cells, while ectopically expressed NR4A2 increases apoptosis and hampers gastrin induced invasion, indicating a tumor suppressor function of NR4A2. Collectively, our results uncover a role of NR4A2 in gastric adenocarcinoma cells, and suggest that both the level and the localization of NR4A2 protein are of importance regarding the cellular responses of these cells.

## Introduction

Gastrin is a gastrointestinal peptide hormone which plays a central role in regulation of gastric acid secretion [1], and in differentiation, maintenance and organization of cells/tissue in the gastric mucosa [2,3]. Beside its role in regulation of normal physiology, gastrin is shown to exert growth promoting impact both in normal and malignant gastrointestinal tissue. Gastrin stimulates proliferation of human gastric and pancreatic cell lines [4-6]. Hypergastrinemia is associated with gastric neuroendocrine tumors (carcinoids) [7] and is found to regulate the expression of anti- and pro-apoptotic genes in both human [8] and rat [9] mucosa. Gastrin mediates its effect via the cholecystokinin-2 receptor (CCK2R), primarily expressed by enterochromaffin-like (ECL) cells, but also reported to be expressed in cancer like colorectal and pancreatic adenocarcinomas [10-12].

The nuclear receptor 4A2 (NR4A2) is a member of the Nurr77 orphan receptor subfamily that comprises NR4A1 (NGIF-B/Nur77), NR4A2 (NOT/Nurr1) and NR4A3 (MINOR/NOR-1). All three family members are immediate early genes induced by physiologic signals including growth factors, hormones and inflammatory cytokines [13,14], and are shown to promote cell proliferation, apoptosis and terminal differentiation in a tissue dependent manner [15,16]. NR4A1 and NR4A3 are silenced in human acute myeloid leukemia (AML), and abrogation of both genes in mice leads to rapid postnatal development of AML [17,18]. NR4A2 is highly expressed in several bladder cancer cell lines and activation of the ligand-binding domain of NR4A2 was demonstrated to induce apoptotic pathways and inhibit growth of bladder cancer established in nude mice [19]. Contrary to the findings in bladder cancer cells, NR4A2 is shown to promote growth of colorectal cancer [20] and to transactivate osteopontin, a direct target of the Wnt/β-catenin pathway associated with colorectal invasion and metastasis [21].

By microarray gene profiling we identified NR4A2 as a gastrin responsive gene in the pancreatic adenocarcinoma cell line AR42J [22]. In this study we examined the role of NR4A2 in the gastric adenocarcinoma cells. Gastrin transiently regulates NR4A2 expression in AGS-G_R_ cells. NR4A2 is known to activate genes via cognate NBRE response elements, and we show that NBRE reporter plasmid is activated upon gastrin treatment. Ectopic expression of the transcriptional repressor ICER (inducible cAMP early repressor) reduces gastrin induced NR4A2 expression as well as transcriptional activation of the NBRE reporter plasmid, indicating that ICER acts as a negative regulator of gastrin induced NR4A2. We also show that gastrin affects the NR4A2 nucleus-cytosol shuttling. We find that ectopic expression of NR4A2 hampers gastrin induced invasion, which indicates a role of NR4A2 in regulating invasive properties of these cells. The molecular mechanisms likely to be involved are gastrin induced changes in the NR4A2 nucleus-cytosolic shuttling and increased apoptosis. Collectively, our study suggests a function of NR4A2 concurrent with a tumor suppressor role in gastric adenocarcinoma cells.

## Materials and Methods

### Cells and reagents

**Figure 1:**
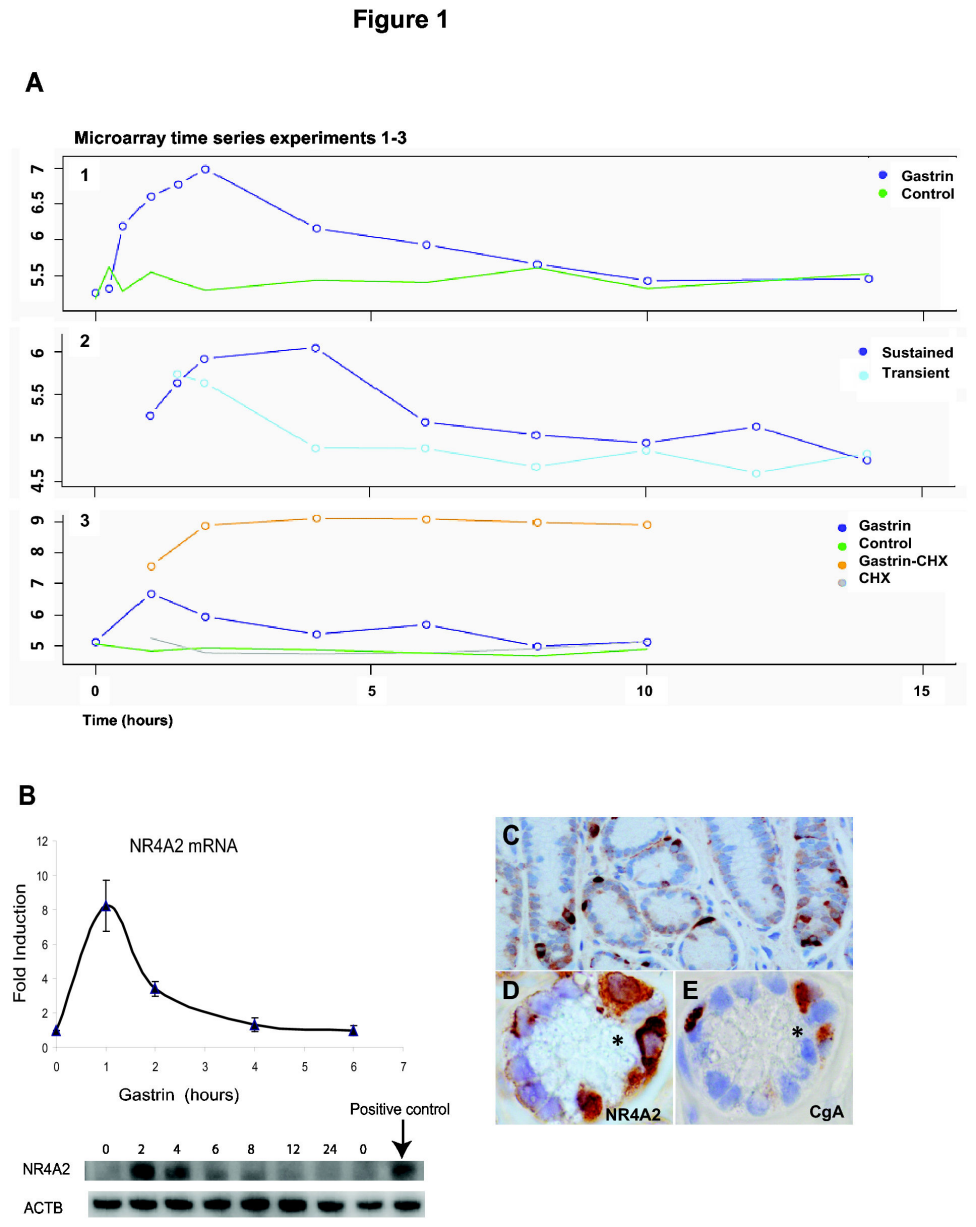
NR4A2 is induced by gastrin. **A**: Temporal profiles of gastrin induced NR4A2 mRNA expression in pancreatic adenocarcinoma cells (AR42J). The panels show data from three independent microarray time series experiments (accession numbers E-MATAB-1268 and GSE32869); and the data points are presented as normalized log_2_-transformed signal intensities. Experiment 1: mRNA expression level for untreated (green line) and sustained gastrin treated (blue line) cells. Experiment 2: mRNA level in cells treated in a sustained mode (14 h of continuous presence of gastrin) and in a transient mode (gastrin was removed after 1 h of treatment). Experiment 3: sustained gastrin treatment was measured in the presence (orange line) and absence (blue line) of cycloheximide (CHX) at 6 different time points between 1 and 10 h. Green and grey lines show mRNA levels in untreated and CHX treated control cells, respectively. All data points are mean of two biological replicates. Gastrin (10 nM) treated and untreated control cells were grown in parallel and harvested (pool of 2-3 technical replicates) at several time points, as indicated in the panels. In experiments with transient *versus* sustained gastrin treatment, the growth medium of untreated and gastrin treated cells was removed 1 h after gastrin treatment; the cells were then washed with serum-free medium before fresh serum-free medium with gastrin (sustained gastrin treated cells) or without gastrin (transiently gastrin treated or untreated cells) was added. In experiments with the protein synthesis inhibitor cycloheximide (CHX), pre-treatments with CHX (10 µg/ml) were initiated 30 min before gastrin (10 nM) was added. **B**: NR4A2 mRNA and protein level in gastrin treated (5 nM) AGS-G_R_ cells. qRT-PCR data shown are mean ± SEM of four biological replicas. Western blot image shows NR4A2 protein. Immunostaining for NR4A2 in normal gastric oxyntic mucosa is shown in panels C-E: Strong NR4A2 immunoreactivity (C) in scattered single cells in normal gastric oxyntic mucosa. Overlap between the cells showing strong NR4A2 immunoreactivity (D) and CgA immunoreactive neuroendocrine cells (E) in serial sections (C at x400 magnification, E and F at x1000 magnification).

Details concerning cultivation and treatment of pancreatic adenocarcinoma AR42J cells for the genome –wide data sets are described elsewhere [22] and in the legend to Figure 1. AGS-G_R_ cells (human gastric adenocarcinoma stable transfected with CCK2R, gift from Prof. Andrea Varro, University of Liverpool) [23] were grown in HAM’S F12 (GIBCO, Invitrogen, Carlsbad, CA) supplemented with 10% FCS and 10 U/ml penicillin-streptomycin and 2µg/ml puromycin (Sigma-Aldrich, St. Louis, MO). AR42J cells (rat pancreatic acinar cell derived with endogenously expressed CCK2R; American Type Culture Collection (ATCC), Rockville, MD) were grown in DMEM (GIBCO, Invitrogen) with 4.5 g/l glucose, 15% FCS, 1 mM sodiumpyruvate, 0.1 mg/ml L-glutamine, 10 U/ml penicillin-streptomycin, and 1 µg/ml fungizone (all GIBCO, Invitrogen). Gastrin (G-17) and cycloheximide (CHX) were purchased from Sigma-Aldrich.

### Expression plasmids

3xNBRE-Luc and pCMX-NR4A2 constructs were kindly provided by Prof Thomas Perlmann, Karolinska Institute, Sweden. pNR4A2-luc, containing the human NR4A2 promoter, was a kind gift from Prof. Marc Montminy [24]. Sequence verification of the plasmid identified -128 to +154 of the NR4A2 promoter, including the CRE element. pCONTROL-luc were obtained from Panomics (CA, USA). NR4A2-EGFP was a kind gift from Prof. Evely Murphy, University College Dublin, Ireland [25]. pICER IIy and pICER I were constructed via homologous recombination of ICER IIy or ICER I containing pDONR201 plasmid [26] and pEF5/FRT/V5-DEST (Invitrogen). Zfp36l1 (Berg36) entry clone (Berg36 ORF Express Shuttle Clone) was ordered from GeneCopoeia (USA), and pZfp36l1-DEST was constructed by homologous recombination with entry clone and pDEST26 (Invitrogen). pCONTROL vectors were constructed by restriction cutting in *att* sites in pEF5/FRT/V5-DEST with EcoRV, and in pDEST26 with BsrGI, followed by re-ligation of *att* sites, thereby removing the insert between the *att* sites. pEF5/FRT/V5/GW-CAT was purchased from Invitrogen.

### Transient transfection and gastrin treatment of cells

AGS-G_R_ cells (5.0 x 10^5^/well) were plated in 6-well plates and transfected after 24 h with 2.5 µg plasmid and 12.5 µl Metafectene PRO transfection reagent (Biontex Laboratories GmbH, Martinsried, Germany) per well. 24 h after transfection, cells were serum starved for 24 h before treatment with gastrin as indicated in figure legends. Overexpression was verified by qRT-PCR and Western blotting (Figure S2C/D).

### siRNA

siRNA-ICER (Qiagen) was designed targeting sites within human ICER: 5’- CAUUAUGGCUGUAACUGGATT-3’, and annealed as described previously [26]. siNR4A2, siRNAs siCONTROL#1, siCONTROL#2 and siGAPDH were obtained from Ambion (Austin, TX). The siCONTROL-pool, ON-TARGET plus Non-Targeting Pool, were obtained from Dharmacon (Lafayette, CO). Downregulation of NR4A2 mRNA and protein was verified by qPCR and Western blotting (Figure S2A/B).

### Genome-wide gene time series expression analysis on Illumina Expression Bead Chips

RNA amplifications and hybridization were performed at the NTNU Genomics Core Facilely (GCF), as previously described Selvik [22]. The data was normalised by *loess* adjustment within time points and average quantile normalised between time points. The data was analysed using the *Limma* (ver. 3.12.1) Bioconductor package [27]. The microarray data were prepared according to minimum information about a microarray experiment (MIAME) recommendations [28] and deposited in the Array Express [29]. Detailed information about the microarray designs and raw data files from the experiments are accessible by use of these accession numbers: GSE32869, and E-MTAB-1268 (Illumina platform).

### Reporter gene assay

Cells (1.5 × 10^4^/well) were plated in 96-well plates 24 h before transfection. Transfection was carried out using Metafectene™ PRO in 5:1 reagent to plasmid ratio, 84 ng plasmid and phRL-null (Promega, Madison, WI) (1:50). The transfection mixture was added to cells 24 h prior to gastrin treatment. Cells were incubated for additional 4 or 6 h, following lysis in 20 µl Promega lysis buffer (Madison, WI). For co-transfections of plasmid and siRNA, 1.2 x 10^4^ cells were plated in 96-well plates, the next day transfected with siRNA to a final concentration of 20 nM using the RNAiMAX reagent (Invitrogen). After 24 h, cells were transfected with plasmid as described above. Luciferase activity was measured using Dual Luciferase kit (Promega), and Wallac 1420 Victor^3^ plate reader (PerkinElmer, Boston, MA). In all experiments, firefly luciferase activity was normalized to Renilla luciferase activity.

### cDNA synthesis and quantitative real-time PCR (qRT-PCR)

Total RNA was extracted using RNeasy Mini Kit (Qiagen, Germantown, MD). RNA integrity, quality and quantity were evaluated by UV fiberoptic spectrophotometer (Nanodrop Technologies, Rockland, DE). cDNA synthesis was performed with 1 µg total RNA in a 20 µl reaction using the REVERSE-IT 1^st^ Strand Synthesis Kit (ABgene, UK). After synthesis, cDNA was diluted 1:2 with RNase-free water. qRT-PCR was performed with 2.5 µl cDNA in 25 µl reaction mix using ABsolute QPCR SYBR Green Mix (ABgene). Quantitative PCR thermal cycling program: 15 min at 95°C, 40 thermal cycles of 15 s at 95°C, 20 s at 60°C and 40 s at 72°C. The primer sequences used for qRT-PCR analyses are shown in Table S1. PCR samples were run in triplicate and the average used for further quantification. The relative expression ratios were calculated using Pfaffl method [30], or the ∆∆Ct-method [31] and individual expression values were normalized by comparison with β-actin or GAPDH.

### Western Blots

Cells were harvested in 100 µl RIPA (Thermo Scientific, Rockford, USA). Blotting, washing and antibody incubation were performed as previously described [32]. Binding of secondary antibodies was visualized by the Super Signal West Femto Maximum Sensitivity Substrate (Pierce, Thermo Scientific, Rockford, IL) and Kodak Image Station 2000R (Kodak, Pittsburgh, PA). The following antibodies were used: anti-NR4A2 from Santa Cruz Biotechnology (Santa Cruz, CA) and Abcam (Cambridge, UK); HRP-conjugated goat anti-rabbit IgG (Cell Signaling, Beverly, MA), mouse monoclonal to beta actin (Abcam), polyclonal HRP-conjugated goat anti-mouse IgG (Dako, Glostrup, Denmark).

### Immunohistochemistry

Sections for immunohistochemistry were taken from formalin fixed paraffin embedded biopsies from our gastric carcinoma biobank, containing both intestinal and diffuse type cancers classified according to Laurén and normal gastric oxyntic mucosa from patients with no evidence of gastric neoplasm that underwent gastroscopy due to dyspeptic complaints (approval Regional Committee for Medical Research Ethics No 018-02). Serial sections were mounted as mirrored. Before immunostaining, the sections (4 µm) were deparaffinised, rehydrated in graded solutions of ethanol and blocked of endogenous peroxidase activity in 3% H_2_O_2_ for 10 min. Antigen retrieval was achieved by boiling in citrate-buffer pH 6.0 for 15 min. NR4A2 was detected using monoclonal anti-NR4A2 (Abcam) (dilution 1:150) and incubation at 4°C overnight. Neuroendocrine cells were detected using monoclonal anti-chromogranin A (CgA) (Dako) (dilution 1:4000) and incubation at 4°C overnight. The immunoreactions were visualized using the rabbit/mouse EnVision-HRP and DAB+ kit (Dako). Counterstaining was done with hematoxylin. Identical concentration of an isotype equivalent antibody from non-immunized animals (mouse IgG2a) (Dako) was used as negative control.

### Immunocytochemical staining and confocal microscopy

Cells (2.0 x 10^4^/well in 200 µl medium with 10% FBS) were seeded on Lab-Tek™ Chambered Coverglass with 8 wells (NUNC, Thermo Scientific, Rockford, IL) and transfected with NR4A2-EGFP. After cultivation for 24 h, cells were serum starved for 24 h and then treated with 5 nM gastrin for 0-60 min. Cells were fixed (4% paraformaldehyde in PBS) for 10 min, washed (PBS x 2) and permeabilized (ice-cold MeOH) for 10 min on ice and washed (PBS x 2). DNA was stained with Draq-5 (1:1000) for 7 min, washed and stored at 4°C over night before confocal microscopy. Confocal microscopy studies were performed with a Zeiss Axiovert 100-M inverted microscope equipped with an LSM 510 laser-scanning unit and a 1.4 numerical aperture ×63 Plan-Apochromat oil immersion objective. To minimize photobleaching, laser power was typically 20% under maximum, and the pinhole was set to 0.8–1.2. Multitracking was used for dual color imaging. The Zeiss LSM Image browser version 4 was used for acquisition, and processing was completed using Adobe Illustrator CS5.

### FRAP analyses

Fluorescence recovery after photobleaching (FRAP) analysis was performed 24 h after transient transfection of AGS-G_R_ cells (0.2 x 10^6^ cells/24mm Petri dish) with 2 µg NR4A2-EGFP and 6 µl Metafectene PRO. Gastrin (10nM) was added and the cells left in the incubator for 20 min. Confocal microscopy was performed with Zeiss LSM 510 Meta Live using a 63X/ 1.4 oil DIC. The settings were configured to produce ten pre-bleach images followed by bleaching with the 488nm line of a 50-mW argon laser operating at 100% laser power. A fluorescence image of single z sections with an optical splice of 0.7 µm was used. The region of interest (ROI) used for bleaching was a circle with 2.5 µm radius. We used a speed of 200 iterations and the bleach time was 7.2 sec. Subsequent imaging continued at the pre bleach speed until 80 sec was reached. Fluorescence recovery was calculated using Sigma plot (Table S2). The confocal imaging was performed at the Cellular & Molecular Imaging Core Facility, Norwegian University of Science and Technology.

### Flow Cytometry

AGS-G_R_ cells were plated in 6-well plates (3 x 10^5^ cells/well). The following day, cells were transfected with 2.5 µg NR4A2-EGFP or control plasmid H3.1-GFP (kind gift from Prof. Terje Johansen, University of Tromsø, Norway) using Metafectene PRO. The next day cells were transferred to serum-free medium. 48 h after transfection, cells were detached from culture plates by Accutase (Sigma-Aldrich) treatment for further processing. The extent of apoptosis was measured using annexin V Alexa Fluor 647 conjugate (Invitrogen). The cells were incubated with annexin V in binding buffer for 1 h, and analyzed using an LSRII flow cytometer (BD Biosciences). The cells were first gated for the absence or presence of EGFP-fluorescence, and then the two populations were further analyzed for apoptosis using annexin V Alexa Fluor 647. Cells positive for annexin V were considered as apoptotic cells. Data were analyzed with FlowJo 7.6 software (Tree Star Inc., Ashland, Oregon, USA).

### Migration assay

The xCELLigence® DP system (Roche Applied Science, Germany) was used for measurement of migration. This system utilizes specialized culture plates that contain gold electrode arrays beneath the bottom of individual wells (CIM plates). Cellular contact with the electrode surfaces increases the impedance across the electrodes. This impedance value is measured by the DP system and is reported in the dimensionless unit of cell index. AGS-G_R_ cells (3.5 x 10^5^/well) were seeded in 6-well plates. After 24 h the cells were transfected with siRNA for 24 h, subsequently serum starved for 24 h and then trypsinated, followed by reseeding (4.0 x 10^4^ cells/well) in CIM-Plate 16 (Roche Applied Science). The plate was placed on the Real-time xCELLigence Cell Analyzer platform at 37°C to measure the migration index for the duration of the experiments. 1 nM gastrin was used as attractant. Cell migration was monitored every 15 min on a RTCA DP instrument for 24 h. Data analysis was carried out using RTCA Software 1.2 (Roche Applied Science).

### Invasion assay

48 hours after transfection, invasion assay were performed in 24-well plates containing 8-µm pore Matrigel-coated inserts according to the manufacturer’s instructions (Becton Dickinson, Bedford, MA). AGS-G_R_ cells (4.0 x 10^4^ cells/well) in 0.5 ml serum-free medium were plated in the insert with or without addition of gastrin (0.3 nM) for 24 h. Cells invading the lower surface of the membrane were stained with Reastain Quick-Diff reagents (Reagena, Finland). The total cells in 5 fields per membrane were counted, and the mean of 3 membranes per experiment was calculated.

### Statistical analysis

qRT-PCR data were statistically analyzed for significant differences using REST (relative expression software tool) [33]. Reporter gene data which include several biological experiments were analyzed using student two-tailed t-test assuming unequal variance. Data were considered significant at p<0.05, unless otherwise stated.

## Results

### NR4A2 expression is activated by gastrin

Genome-wide time series experiments identified *NR4A2* as a gastrin responsive gene in the pancreatic adenocarcinoma cell line AR42J (Figure 1A). The mRNA expression was transient with peak expression at 2 h, followed by decrease to baseline after ~6-8 h of gastrin treatment (Figure 1A, panel1). Genome-wide microarray time series analysis was also used to identify genes that are affected by the duration of gastrin treatment in adenocarcinoma cells [22]. As shown in Figure 1A, panel 2, the expression of NR4A2 was higher and more prolonged in cells treated in a sustained mode (gastrin present for 14 h) than in a transient mode (gastrin removed after 1 h). To establish the role of new protein synthesis in gastrin-mediated regulation of *NR4A2* transcript levels, we analyzed gastrin treated AR42J cells in the presence of the translational inhibitor cycloheximide (CHX). We found that the initial increase in transcript levels occurs in the presence of CHX, demonstrating that *NR4A2* is a primary gastrin responsive gene (Figure 1A, panel 3). The decline of NR4A2 transcript levels is abolished in the presence of CHX, indicating that the transient nature of the gastrin induced *NR4A2* transcripts is dependent upon *de novo* protein synthesis of a transcription inhibitor or of proteins that reduce mRNA stability. Quantitative real-time PCR confirmed that gastrin induced transient expression of NR4A2 mRNA, followed by decrease to baseline after ~6 h of stimulation; and that NR4A2 mRNA expression was sustained when protein synthesis was inhibited by CHX (Figure S1).

We further examined the role of NR4A2 in gastrin induced responses by employing the gastric adenocarcinoma cell line AGS-G_R_ Gastrin induced a ~8-fold induction of NR4A2 mRNA in AGS-G_R_ cells, followed by a rapid decrease to baseline after ~4 h of stimulation (Figure 1B). The NR4A2 protein expression peaks at 2 h and displays a rapid decrease after 6 h, in agreement with what is reported for other cells [34].

The expression pattern of endogenous NR4A2 was also observed *in vivo* by immunohistochemistry. In normal gastric oxyntic mucosa (n=4) there was strong immunoreactivity in small, scattered single cells predominantly in the basal part and situated between the other epithelial cells (Figure 1C). This appearance is suggestive of neuroendocrine cells, and this was confirmed by overlap in serial staining using antibody against the neuroendocrine marker CgA (Figure 1D-E). NR4A2 was most strongly expressed in cytoplasm of these cells, but also nuclear staining was observed. In addition there was weaker immunoreactivity in other epithelial cells of the mucosa (Figure 1C). Since the neuroendocrine cell population in oxyntic mucosa is dominated by the ECL cell, which is known to possess the CCK2R and to be the main gastrin responsive epithelial cell [35], this supports our results showing that NR4A2 expression is activated by gastrin.

### Gastrin-induced NR4A2 activates NBRE promoter elements

**Figure 2:**
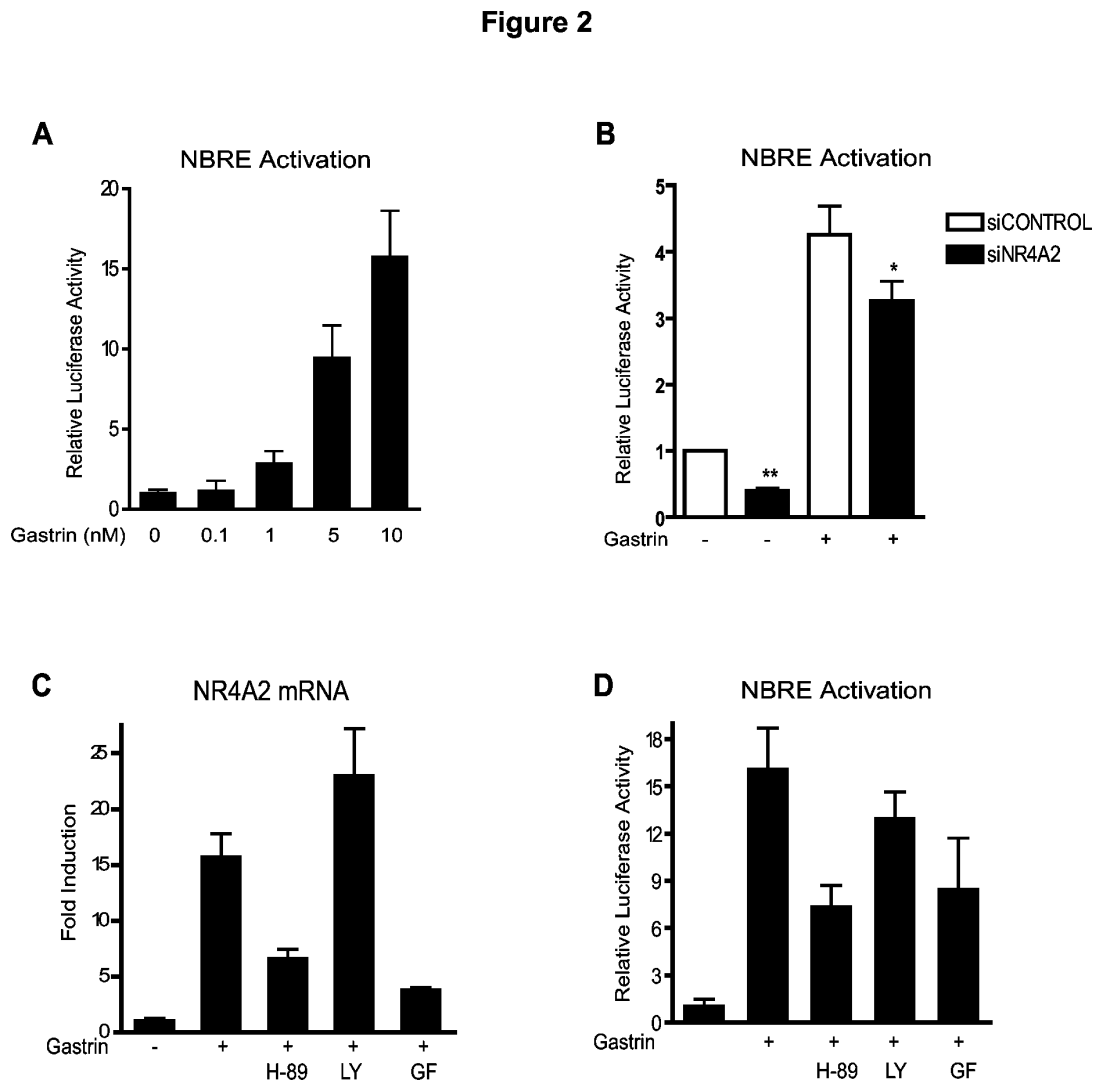
NR4A2 activates NBRE promoter elements. **A**: Gastrin-induced NBRE-luc activation. Data represent one of two biological replicas. **B**: The effect of NR4A2 siRNA on gastrin-induced NBRE activation. Data represent mean ± SEM of four biological replicas (** p<0.01, * p=0.1). **C-D**: Effect of specific inhibitors of PKA (H-89, 10µM), PI3K (LY 294002, 10µM) or PKC (GF 109203x, 3.5µM) on (**C**) gastrin-induced NR4A2 gene expression and (**D**) gastrin-induced NBRE activation. Data represent one of three biological replicas; mean ± SD of six technical replicas.

NR4A2 is known to activate target genes via the cognate NBRE response element [36]. We wanted to determine whether gastrin could affect NBRE regulated genes and thus measured the NBRE reporter gene activity in gastrin treated AGS-G_R_ cells. Our results show that gastrin activates NBRE-driven gene expression in a dose-dependent manner (Figure 2A). To verify that this gastrin response is mediated via NR4A2, NBRE reporter gene activity was measured in gastrin treated cells transfected with siRNA targeting NR4A2. We demonstrate that siNR4A2 significantly reduces gastrin-mediated activation of NBRE (Figure 2B), suggesting that NR4A2-activated gene expression plays a role in gastrin mediated responses.

The cellular effect of gastrin is transmitted via the Gα_q/11_ protein-coupled CCK2R and known to target a cascade of intracellular mediators including protein kinase C (PKC), phosphoinositide 3-kinase (PI 3-kinase), mitogen-activated protein kinases (MAPKs) and protein kinase A (PKA) [37-40]. Hence we examined the signaling pathways involved in gastrin-mediated NR4A2 activation. Gastrin-induced NR4A2 gene expression in AGS-G_R_ cells was significantly reduced by inhibitors of PKA or PKC, but not by the PI3K inhibitor (Figure 2C). This was some unexpected since gastrin mediated signaling is reported to phosphorylate PKB/Akt [41]. However, Western blot experiments demonstrated a constitutive phosphorylation of PKB/Akt Ser-473 in AGS-G_R_ cells (data not shown), which likely explain why we did not observe any effect of the LY294002 inhibitor. The NBRE reporter gene experiments demonstrated that PKA and PKC signaling pathways both participate in gastrin mediated NBRE transcriptional activation (Figure 2D). Taken together, the results indicate that gastrin induces NR4A2 gene expression and NBRE target gene activation via PKA and PKC signaling pathways.

### NR4A2 is negatively regulated by gastrin-induced proteins

**Figure 3:**
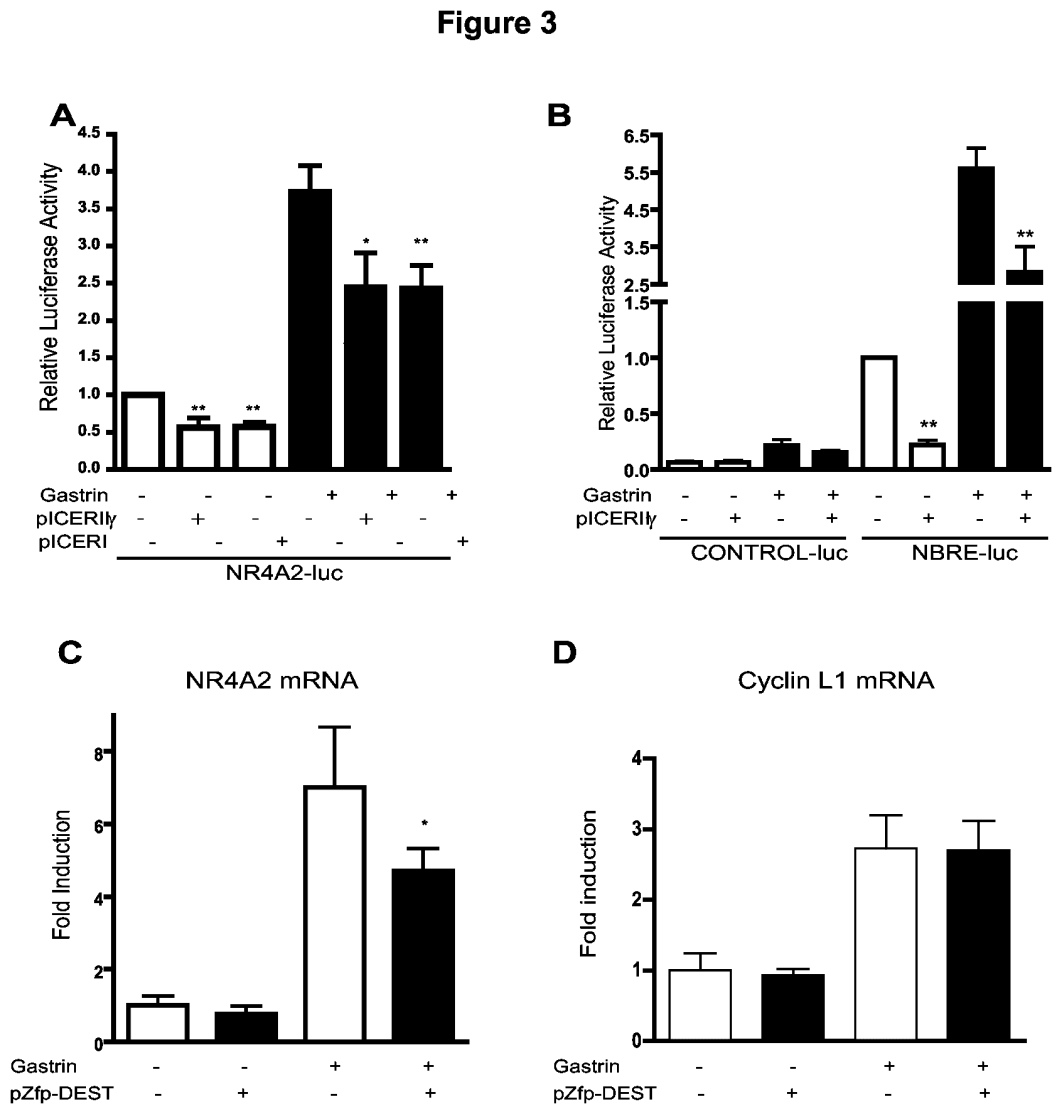
Negative regulation of gastrin-induced NR4A2 expression. **A**: AGS-G_R_ cells transfected with NR4A2-luc and ICER expression plasmids or empty vector. Cells were treated with gastrin for 6 h prior to measurement of NR4A2 activity. Data shown represent mean ± SEM of five biological replicas (** p<0.03, * p = 0.06). **B**: AGS-G_R_ cells transfected with NBRE-luc and ICER expression plasmid or empty vector and treated with gastrin for 4 h prior to measurement of NBRE activity. Data shown represent mean ± SEM of four biological replicas (** p<0.03). **C**: AGS-G_R_ cells were transfected with pZfp36l1 expression plasmid or empty vector and treated with gastrin (5 nM) NR4A2 mRNA expression was measured by qRT-PCR. Data shown represent one of three biological replicas; mean ± SD of three technical replicas is shown. **D**: Cyclin L1 represents one of three control genes examined.

We have previously shown that gastrin induces expression of Inducible cAMP early repressor (ICER) [42], and that ICER represses gastrin-induced genes with CRE promoter elements [26,32]. The promoter region of the *NR4A2* gene comprises CRE regulatory elements [43,44]. Thus, it was of interest to investigate whether ICER could modulate gastrin-induced NR4A2 expression and with this constitute a gastrin induced negative feedback mechanism. AGS-G_R_ cells were transfected with ICER I or ICER IIγ expression plasmids together with a reporter plasmid for NR4A2 promoter activity; NR4A2-luc. Our results show that ectopically expressed ICER significantly reduces gastrin-induced NR4A2 gene expression (Figure 3A). In addition, we find that ICER expression reduces NBRE reporter gene activity by ~ 50% in gastrin treated cells (Figure 3B). ICER also affects NBRE activity in untreated cells. Our results demonstrate that the role of ICER as a negative feedback regulator of gastrin responses involves both downregulation of NR4A2 gene expression and repression of gastrin-induced NBRE-regulated genes.

*NR4A2* possesses AU-rich elements (AREs) in its 3`-untranslated region (3'-UTR)(http://rna.tbi.univie.ac.at/cgi-bin/AREsite.cgi), and Zinc finger protein 36, C3H1 type-like 1 (Zfp36l1) is suggested to participate in the degradation of short-lived, inducible mRNAs by binding to AREs [45,46]. We found that gastrin induced *Zfp36l1* expression in AR42J cells in two independent microarray time series (E-MATAB-123 (cDNA microarrays) and GSE32869 (Illumina)) and therefore examined whether Zfp36l1 would influence the NR4A2 mRNA levels. AGS-G_R_ cells were transfected with Zfp36l1 expression plasmid, and the amount of NR4A2 mRNA was assessed by qRT-PCR. Gastrin-treated cells with ectopic expression of Zfp36l1 exhibited significantly reduced levels of NR4A2 transcripts (Figure 3C). No effect of Zfp36l1 was observed in the control experiments, by qRT-PCR measurement of the expression of non-ARE genes like *CyclinL1* (Figure 3D) and *Ywhag* (data not shown). We conclude that NR4A2 transcript levels are negatively regulated by at least two different gastrin induced mechanisms: ICER represses the transcription, while Zfp36l1 reduces NR4A2 mRNA levels by affecting its degradation.

### Gastrin facilitates change in the nucleus-cytosol shuttling

**Figure 4:**
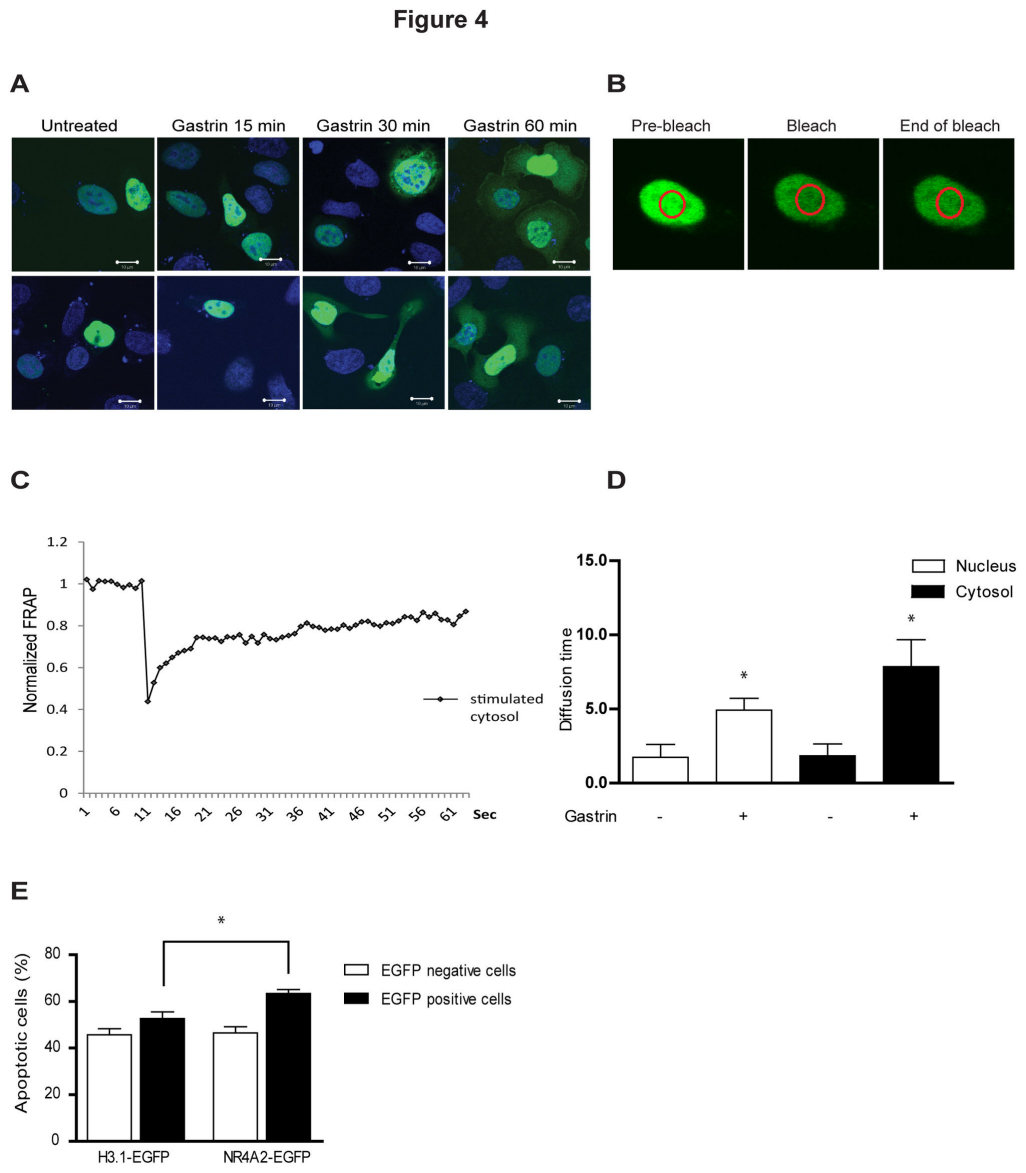
Gastrin treatment influences nucleus-cytosolic shuttling of NR4A2. **A**: Intracellular localization of NR4A2 protein in response to gastrin treatment. AGS-G_R_ cells transfected with pNR4A2-EGFP. **B**: Images of gastrin treated (10 nM) AGS-G_R_ cells expressing pNR4A2-EGFP before, during and after bleaching of a nucleus area for 7.5 sec. The circle indicates the area of the bleach spot. **C**: Normalized FRAP curve for the cytosol of gastrin treated AGS-G_R_ cells. **D**: Diffusion time in untreated and gastrin treated nucleus and cytosol. **E**: To determine cell viability, cells were transfected with pNR4A2-EGFP or a control plasmid (pH3.1-EGFP). After 48 h AGS-G_R_ cells were detached by Accutase treatment, labeled with annexin V Alexa Fluor 647 and analyzed by flow cytometry. Annexin-V positive cells were considered as apoptotic. Results are shown as % apoptotic cells of the total number of counted EGFP positive or EGFP negative cells. Data are representative of three biological replicas; mean ± SD of three technical replicas is shown.

Subcellular trafficking of nuclear receptors and the subsequent protein interactions often affect the cellular response. In addition to transactivation functions, NR4As are reported to modulate the activity of other proteins through subcellular translocation and protein-protein interactions [47-51]. Thus it was of interest to establish a putative role of gastrin in modifying the trafficking of NR4A2 proteins. AGS-G_R_ cells were transfected with NR4A2-EGFP and treated with gastrin up to 60 min. We show that gastrin facilitates NR4A2 nucleus-cytosolic translocation (Figure 4A); increasing amount of NR4A2-EGFP was located in the cytosol upon gastrin treatment compared to the control where the better part of NR4A2 is localized in the nucleus. To further explore the protein shuttling, FRAP experiments were included. Normalization curves were used to optimize the bleach parameters. Based on a 2D diffusion model, we fitted the individual FRAP curves using a non-linear regression analysis (Sigmaplot) (Figure 4 B/C) [52,53]. We observed a significant change in the diffusion time comparing gastrin treated *versus* untreated cells, both in the nucleus and the cytosol (Figure 4D). Our results show that NR4A2-EGFP resides for a longer time (i.e. higher diffusion time) in cytosol compared to nucleus in gastrin treated cells. The fact that NR4A2 is present for a longer time period in cytosol compared to nucleus may affect the cellular response. This is analogous to what has been described for NR4A1, where mitochondrial localization is shown to induce apoptosis [48]. Hence, we examined whether apoptosis was induced in AGS-G_R_ cells ectopically expressing NR4A2, using flow cytometry and annexin V Alexa Fluor 647 labeling. We observed ~20% more apoptosis (p<0.05) in cells overexpressing NR4A2 compared to controls (i.e. cells transfected with the control plasmid H3.1-EGFP) (Figure 4E). Treatment with gastrin did not influence the number of apoptotic cells in this time period (data not shown).

### NR4A2 suppresses gastrin-induced migration and invasion

**Figure 5:**
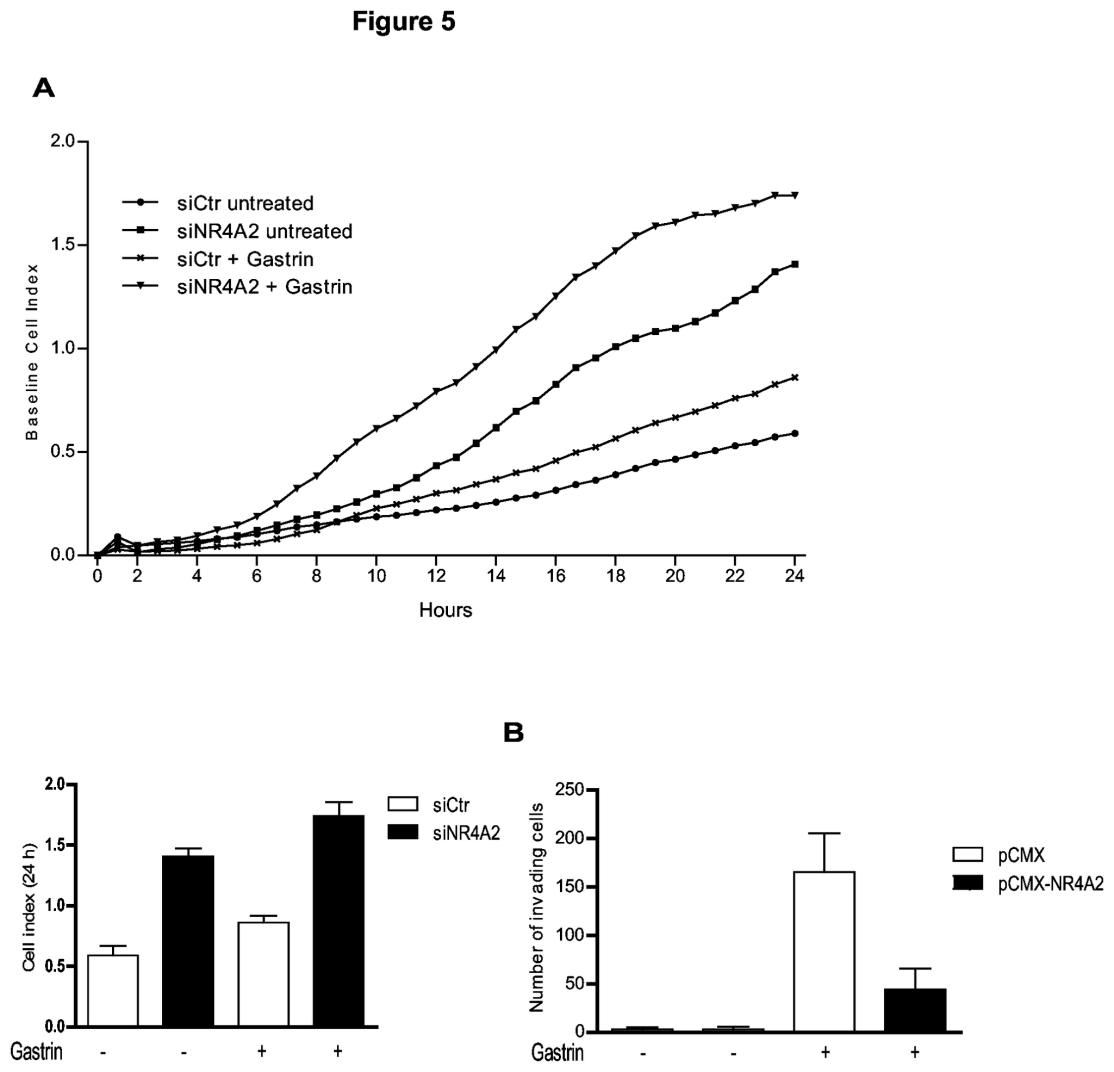
NR4A2 suppresses gastrin-induced migration and invasion. **A**: Real-time cell migration monitored (0-24 h) in AGS-G_R_ cells transfected with siNR4A2 or siCtr, with or without gastrin treatment (10 nM). Results show one representative of three biological replicas; mean ±SD of three technical replicas. **B**: Invasion assay with AGS-G_R_ cells transfected with pCMX-NR4A2 or pCMX (control) was performed in 24-well plates containing 8-µm pore Matrigel-coated inserts (with or without 0.3 nM gastrin). Cells invading the lower surface of the membrane were stained with Reastain Quick-Diff reagents and total numbers of cells in 5 fields per membrane were counted. The mean of three independent experiments is shown.

Little is known about the molecular mechanisms involved in NR4A2 regulation, and conflicting data exists concerning its role in cancer. However, NR4A2 has been characterized as a putative tumor suppressor protein in gastric cancer, being down-regulated both in primary gastric cancers and in synchronous liver metastases [54]. Thus it was of interest to examine whether NR4A2 would influence gastrin induced migration and invasion in our gastric adenocarcinoma cells. AGS-G_R_ cells were transfected with siRNA targeting NR4A2 and migration assessed using real-time cell monitoring assay (xCELLigence technology). As shown in Figure 5A, NR4A2 knock-down by itself resulted in a significant increase of migration and gastrin treatment further enhanced this effect. Next we determined the importance of NR4A2 in invasion, now using AGS-G_R_ cells with ectopically expressed NR4A2. We show that ectopic expression of NR4A2 dramatically reduces the number of invading cells as a consequence of gastrin treatment (Figure 5B). Collectively, these results suggest that high level of NR4A2 hampers the migratory potential of AGS-G_R_ cells.

### NR4A2 protein is expressed in tumor cells in gastric adenocarcinomas

**Figure 6:**
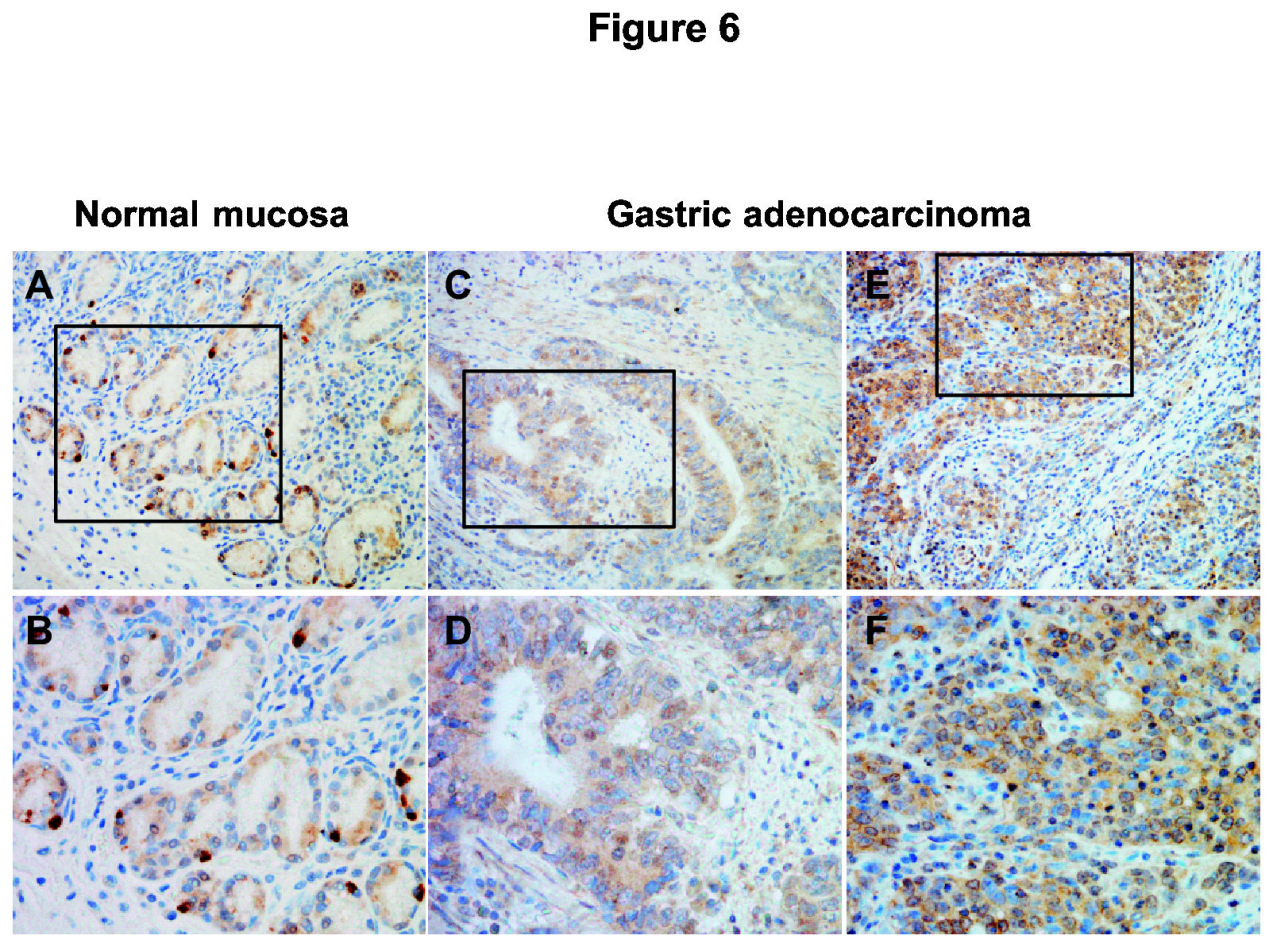
Immunostaining of NR4A2 in gastric adenocarcinoma. **A-B**: NR4A2 immunoreactivity in normal oxyntic mucosa showing strong intensity in scattered single cells (neuroendocrine cells) and weaker staining intensity in the other epithelial cells. **C-F**: NR4A2 immunoreactivity in gastric adenocarcinomas of intestinal (C-D) and diffuse (E-F) type, showing a general staining in tumor cells with mixed nuclear or cytoplasmic localization and variable intensities. (A, C, E at x200 magnification, with boxes representing B, D and F at x400 magnification).

To further substantiate the relevance of our findings *in vivo*, we analyzed the protein expression of NR4A2 in a small collection of gastric adenocarcinomas (intestinal n=3, diffuse n=3) by immunohistochemistry (Figure 6A-F). Compared to normal gastric oxyntic mucosa the expression pattern of NR4A2 seemed to be changed in both cancer types. There was not observed any strong staining specifically in single cells. The pattern was rather dominated by a general expression of NR4A2 in tumor cells, showing mixed nuclear or cytoplasmic localization and variable intensities. Some weak staining was also seen in mesenchymal cells. No differences regarding the type of gastric adenocarcinoma or nucleus *versus* cytosolic localization could be read from this small cohort. Our findings are in accordance with the OncoMine database (https://www.oncomine.org), where *NR4A2* gene expression is shown to vary both within and among subtypes of gastric adenocarcinomas; being both higher and lower expressed compared to normal controls.

## Discussion

In the present study we address the function of the orphan nuclear receptor NR4A2 in gastrin regulated responses. Our principal findings are that gastrin regulates NR4A2 expression and activity in gastric adenocarcinoma AGS-G_R_ cells. The regulation involves gastrin induced nucleus-cytosolic shuttling of NR4A2. We find that sustained expression of NR4A2 inhibits gastrin induced invasiveness, which is congruent with a tumor suppressor function of NR4A2 in AGS-G_R_ cells. This is in contrast to a transient expression of NR4A2 which does not affect gastrin induced invasiveness or proliferation [55,56], indicating that a threshold level of NR4A2 is of vital importance for the cellular decision towards migration/invasion. The gastrin induced regulation of NR4A2 was further substantiated *in vivo* by strong NR4A2 expression in the gastrin responsive neuroendocrine ECL cells in normal gastric oxyntic mucosa.

Gastrin is known to promote proliferation, migration and invasion of AGS-G_R_ cells [55-57]. Thus, the transient induction of NR4A2 by gastrin may play a role in the gastrin induced migration and invasion of these cells. A putative cross talk between NR4A2 and the Wnt signaling pathway has been suggested [49], involving NR4A2 cytoplasmic translocation and de-repression of transcription upon beta-catenin treatment. However, in contrast to transient induction of NR4A2, we show that sustained (i.e. ectopic) expression of NR4A2 increases apoptosis and hampers the invasiveness of AGS-G_R_ cells. This may suggest that some threshold effect of NR4A2 exists when it comes to its influence on apoptosis of gastric adenocarcinoma cells, corresponding to what is found in the breast cancer study by Llopis et al [58]. Low expression level of NR4A2 in AGS-G_R_ cells is supportive with migration and invasion (untreated AGS-G_R_ cells show low levels of NR4A2 proteins, Figure 1B), while high or sustained level of NR4A2 reduces the invasiveness of the cells through distinct mechanisms, which also involves apoptosis. We speculate that the increased apoptosis of AGS-G_R_ cells might be due to NR4A2 mediated modulation of proteins through protein-protein interactions in cytosol, and/or NR4A2 localization/association with mitochondria analogous to what has been described for NR4A1 [48]. Interestingly, nucleus to cytosol trafficking of NR4A1 is suggested to be the molecular switch that dislodges the Bcl-2 BH4 domain, exposing its BH3 domain, which in turn blocks the activity of anti-apoptotic Bcl-X(L) [48]. Whether NR4A2 is involved in a corresponding mechanism in gastric adenocarcinoma cells is not known. Taken together, we advocate that high and sustained level of NR4A2 in AGS-G_R_ cells reduces migration/invasion partly by promoting apoptosis, while transient expression of NR4A2 does not seems to affect such mechanisms.

NR4A2 is characterized as a hub gene [54], a term initially used to describe central proteins of transcriptional networks [59]. Hub proteins may regulate quite different biological processes since they interact with several proteins and represent important regulatory nodes in biological networks. In a recent study investigating the expression of NR4A2 in breast cancer, the authors concluded that NR4A2 expression in breast is commensurate with a normal and terminally differentiated epithelial phenotype, whereas silencing or dysregulation of NR4A2 probably plays a role in oncogenic transformation of breast epithelial cells [58]. In accordance with this, we also observed changed NR4A2 expression in gastric adenocarcinomas, seemingly being dominated by a general expression in tumor cells compared to the mainly strong NR4A2 expression in the gastrin responsive neuroendocrine ECL cells in normal mucosa. In tumor cells the expression showed variable intensities which is in agreement with our own data and the data from OncoMine (https://www.oncomine.org). Our finding in normal mucosa is in contrast to Chang et al [54] showing primarily mesenchymal expression of NR4A2 in normal mucosa, with a change to stronger epithelial expression in primary gastric cancers and a further nearly loss of expression in paired liver metastasis. In addition to the dichotomous behavior of NR4A2, differences in antibody specificities could also explain such differences. In a study from Holla et al [20] intestinal epithelium from Apc^-/+^ mouse adenomas and sporadic colorectal carcinomas exhibit increased NR4A2 expression relative to matched normal mucosa. However, the mechanisms elucidated in this study indicated that NR4A2 was important for PGE2-mediated regulation of apoptosis, and thus is likely to mirror mechanisms such as inflammatory signaling pathways, which are known to play a prominent role in colorectal cancer. Taken together, the partly conflicting results published so far, probably reflect both tissue- and cell specific differences in addition to a biphasic role of NR4A2.

In this study we throw light on the dichotomous role of NR4A2 in cancer. We conclude that gastrin induced NR4A2 expression and transactivation play an important role in gastric adenocarcinoma cells. The amount of NR4A2 protein and/or the lack of negative feedback regulation may switch the cellular response. A better understanding of gastrin–NR4A2 regulated processes may reveal new strategies to treatment of gastric adenocarcinomas.

## Supporting Information

Figure S1 **NR4A2 mRNA expression in gastrin treated AR42J cells measured by qRT-PCR.** The cells were pre-treated with the protein synthesis inhibitor cycloheximide (CHX) (10 µg/ml) for 30 min before gastrin (10 nM) was added. Data represent one of three biological replicas; mean ± SD of three technical replicas.
(EPS)

Figure S2 **Effect of siNR4A2 and pCMX-NR4A2 expression plasmid.** **A**: qRT-PCR data showing NR4A2 mRNA expression in gastrin treated AGS-G_R_ cells transfected with siNR4A2 or siCtr. **B**: Western blot showing NR4A2 protein in gastrin treated AGS-G_R_ cells transfected with siNR4A2 or siCtr. **C**: qRT-PCR showing NR4A2 mRNA expression in gastrin treated AGS-G_R_ cells transfected with pCMX-NR4A2 or pCMX. **D**: Western blot showing NR4A2 protein in gastrin treated AGS-G_R_ cells transfected with pCMX-NR4A2 or pCMX.
(EPS)

Table S1 **PCR primers.**
(PDF)

Table S2 **Experimental conditions.**
(PDF)
