# Supplementary material for: NR4A2 Is Regulated by Gastrin and Influences Cellular Responses of Gastric Adenocarcinoma Cells
Source: PLoS One. 2013 Sep 27;8(9):e76234. doi: 10.1371/journal.pone.0076234 (PMC3785466; doi:10.1371/journal.pone.0076234)
Supplement: Table S1 — PCR primers. (PDF) [file pone.0076234.s003.pdf]

Supporting Table S1. PCR primers.

Gene Symbol	Species	Sense 5'- 3'	Antisense 5'- 3'	Reference
CyclinL1	human	GCAGGTGTTGTTTCATCGTT	CGGAGGTGGTGGAATACATTA	
GAPDH	human	GAAGGTGAAGGTCGGAGTC	GAAGATGGTGATGGGATTTC	
β2M	human	GAATTCACCCCCACTGAAAA	AGCAAGCAAGCAGAATTTGG	
NR4A2	human	GTCTCAGCTGCTCGACACG	TTTTGCACTGTGCGCTTAAA	[1]
NR4A2	rat	CTACGCTTAGCATACAGGTC	TTCCTTGAGCCCGTGTCT	
β-actin	rat	CTGGCTCCTAGCACCATGA	AGCCACCAATCCACACAGA	

1. Holla, V. R.; Mann, J. R.; Shi, Q.;DuBois, R. N. Prostaglandin E2 regulates the nuclear receptor NR4A2 in colorectal cancer. *The Journal of biological chemistry* **2006**, *281*, 2676-2682.
