# Supplementary material for: NR4A2 Is Regulated by Gastrin and Influences Cellular Responses of Gastric Adenocarcinoma Cells
Source: PLoS One. 2013 Sep 27;8(9):e76234. doi: 10.1371/journal.pone.0076234 (PMC3785466; doi:10.1371/journal.pone.0076234)
Supplement: Table S2 — Experimental conditions. (PDF) [file pone.0076234.s004.pdf]

Supporting Table S2. Experimental conditions

**Experimental conditions;** Zeiss LSM 510 Meta Live	
37 °C, 5% CO_2	
Objective	63X/1.4 OIL PLAN APO
Scan mode	xyz
Format	256 x 256
ROI	2.5 μm in diameter
Argon laser power	50 mW, 100% output
Bleaching 488 nm	100% (Zoom in mode)
Imaging 488 nm	5%
Emission range	490-530 nm
Pre-bleach	10 sec
Bleach	7.5 sec
Post-bleach 1	80sec
